# Tracking poliovirus through wastewater: environmental surveillance insights from Haïti (2020–2023)

**DOI:** 10.1128/aem.01179-25

**Published:** 2025-11-18

**Authors:** Hanen Belgasmi-Allen, Jamaica Hill, Stacey Jeffries Miles, Leanna Sayyad, Hala Elahi, Nicole E. Patterson, Ashley Deas, Amelus Heberlienne, Amanda Wilkinson, Everardo Vega, Cara C. Burns, Nancy Gerloff

**Affiliations:** 1 Polio and Picornavirus Branch, Division of Viral Diseases, Centers for Disease Control and Prevention 1242, Atlanta, Georgia, USA; 2 Cherokee Nation Assurance, Contracting agency to the Division of Viral Diseases, Centers for Disease Control and Prevention, Tulsa, Oklahoma, USA; 3 Tanaq Management Services LLC, Contracting agency to the Division of Viral Diseases, Centers for Disease Control and Prevention, Anchorage, Alaska, USA; 4 Division of Epidemiology, Laboratory and Research, Ministère de la Santé Publique et de la Population (Ministry of Public Health and Population (MSPP), Port au Prince, Haïti; 5 Polio Eradication Branch, Global Immunization Division, Centers for Disease Control and Prevention 1242, Atlanta, Georgia, USA; University of Nebraska-Lincoln, Lincoln, Nebraska, USA

**Keywords:** polio eradication, environmental surveillance, vaccine-derived polioviruses, wild poliovirus, enterovirus, Haïti

## Abstract

Environmental surveillance (ES) for poliovirus is a critical component of global eradication efforts, enabling the detection of virus circulation in communities, even in the absence of acute flaccid paralysis (AFP) cases. To monitor poliovirus circulation in a high-risk setting, ES was established in 2016 in Haïti in two cities (Port-au-Prince and Gonaïves) and expanded to five cities, adding Saint Marc, Cap Haïtien, and Port-de-Paix by 2023. Wastewater samples were collected monthly from 13 sites in urban areas and processed at CDC-Atlanta using the concentration and filtration elution (CaFÉ) method developed to concentrate enteroviruses, including polioviruses. Virus isolation was performed using poliovirus-sensitive (L20B) cells and enterovirus-sensitive (RD) cells, followed by molecular detection screening via real-time reverse transcription PCR designed for enterovirus and poliovirus typing. Between 2020 and 2023, sporadic Sabin-like poliovirus vaccine strains of serotypes 1 and 3 were detected, and no wild poliovirus (WPV) or vaccine-derived poliovirus (VDPV) was identified. The overall enterovirus detection rates ranged from 36% to 91% in 2020, 12% to 58% in 2021, 16% to 60% in 2022, and 27% to 73% in 2023, with some detection rates at some sites much lower than the optimal 50% target for global ES sites. Based on the lower detection rate, used as the main quality indicator for optimal ES, two sampling sites were discontinued in 2023 (one in Saint Marc and one in Cap Haïtien). The findings underscore the importance of high-performing ES in high-risk regions to augment AFP surveillance and serve as an early warning indicator of poliovirus circulation.

**IMPORTANCE:**

Poliovirus environmental surveillance (ES) is a critical tool for detecting virus circulation before symptomatic cases of acute flaccid paralysis occur, especially in areas with inadequate surveillance. Haïti, a high-risk country for poliovirus transmission, faced numerous challenges from 2020 to 2023 that impacted ES operations, including political instability, humanitarian crisis, and site closures. This study provides a comprehensive evaluation of ES performance during that period, offering insights into surveillance resilience and sustainability in low-resource settings. The findings are timely and relevant, particularly in the context of the recent circulating vaccine-derived poliovirus type 2 (cVDPV2) outbreaks in Europe and Africa, and contribute to optimizing ES strategies globally to support polio eradication efforts.

## INTRODUCTION

Poliovirus (PV), the etiologic agent of poliomyelitis, is a serotype of the species *Enterovirus C*, within the family of *Picornaviridae* (1). Through support of the Global Polio Eradication Initiative (GPEI), wild PV types 2 (WPV2) and 3 (WPV3) were certified eradicated in 2015 and 2019, respectively (2). However, the remaining WPV serotype, wild PV type 1 (WPV1), remains endemic in Pakistan and Afghanistan and briefly circulated in Malawi and Mozambique in 2021 and 2022 after an importation event (3–6). The Americas became the first World Health Organization (WHO) region certified free of indigenous transmission of the three WPV types in 1994, and more recently, the African region was certified in 2020 (7, 8).

The Sabin PVs contained in the oral polio vaccine (OPV) can be transmitted for prolonged periods of time within a population with sub-optimal immunity to PVs. Reversion can occur within days to weeks after OPV administration through only a few critical nucleotide substitutions, most often in the 5′ untranslated region and select capsid sites in the VP1 region, leading to renewed virulence, becoming known as vaccine-derived PVs (VDPVs) (9–11). VDPVs are defined by greater sequence divergence from OPV virus strains that are >1% divergent (or ≥10 nucleotide [NT] changes, for types 1 and 3) or >0.6% divergent (≥6 NT changes, for type 2) from the corresponding OPV strain in the VP1 gene, reflecting prolonged replication or circulation over months (11, 12). In recent years, VDPVs have become a growing concern for the GPEI, leading to outbreaks of circulating VDPVs (cVDPVs) accounting for an abundance of polio cases (9). In response, the GPEI revised the polio eradication strategy 2022–2026, emphasizing the interruption of cVDPV transmission as a key priority along with the permanent interruption of WPV1 transmission and prevention of reestablishment of PV transmission in polio-free regions (13).

Acute flaccid paralysis (AFP) surveillance remains the cornerstone of PV detection, focusing on children under 15 years of age with sudden-onset muscle weakness or paralysis (14). However, since <1% of PV infections manifest as AFP, this surveillance alone may fail, or be slow, to detect community-wide PV transmission, which often precedes symptomatic cases (15). Given that PVs, including the strains in OPV, replicate in the gastrointestinal tract and are shed in feces, environmental surveillance (ES) has emerged as a crucial complement to AFP surveillance by increasing the sensitivity of PV detection and can be especially useful in settings with suboptimal polio immunization coverage (13–16). ES consists of the periodic collection of sewage or wastewater containing composite fecal material contributed by a population, and the subsequent laboratory analyses that can detect PVs (15).

Globally, ES has successfully complemented AFP surveillance. It has supported early PV detection and outbreak response in countries in Africa, such as Nigeria and the Democratic Republic of Congo (17, 18). ES has demonstrated the ability to identify PV circulation in the absence of AFP cases in Israel, where a WPV1 was detected in sewage samples in 2013 without any associated AFP cases, and it also helped AFP surveillance systems in endemic or conflict-affected countries (i.e., Afghanistan, Pakistan, Yemen, and Somalia) and with PV importation monitoring in Europe (18–24). The value of ES was underscored during the 2022 cVDPV2 outbreak in New York State, where the first U.S. paralytic polio case in nearly a decade triggered extensive wastewater monitoring, confirming PV circulation across several counties (25–27). The location and timeline of cVDPV2 detections guided emergency vaccination efforts and later confirmed the cessation of cVDPV2 transmission, defining the end of the outbreak (27, 28).

Recognizing the risks of PV circulation through importation and the emergence of cVDPV in countries with suboptimal polio immunization coverage in the Americas, the Pan American Health Organization (PAHO) began to consider the establishment of ES in the region (29).

Experiences from some countries in the Americas further support the value of ES. In Guatemala, ES initiated in 2018 contributed to an early VDPV detection, triggering targeted investigations for any associated AFP cases and vaccination campaigns (30). In Brazil, WPV1 was detected in sewage during the 2014 FIFA World Cup, with genetic links to the strain associated with an outbreak in Equatorial Guinea (West Africa), demonstrating ES’s utility in detecting PV importation into a non-endemic region (31).

Haïti, located in the Hispaniola Island, characterized by persistently low polio immunization coverage, poor sanitation infrastructure, and a cVDPV1 outbreak in 2000–2001, has been among PAHO’s priority countries for ES implementation (32–34). Although a brief ES effort was conducted in Port-au-Prince from November to December 2000 in response to the cVDPV1 outbreak, Haïti formally launched its ES for PVs program in 2016 to complement its AFP surveillance system (32, 35, 36).

This report presents findings from the continuation of ES for PVs in Haïti from 2020 to 2023. It details sample collection, laboratory methods, and molecular analyses, along with a performance assessment of ES sites using annual enterovirus detection rates as a key indicator for site retention or termination (37). The report also discusses operational challenges, programmatic limitations, and broader implications of sustaining ES in Haïti.

## MATERIALS AND METHODS

Environmental surveillance for PVs is a surveillance activity conducted by the Haïtian Ministère de la Santé Publique et de la Population (Ministry of Public Health and Population [MSPP]) with laboratory and technical support from CDC-Atlanta.

### Sampling site selection

**Fig 1:**
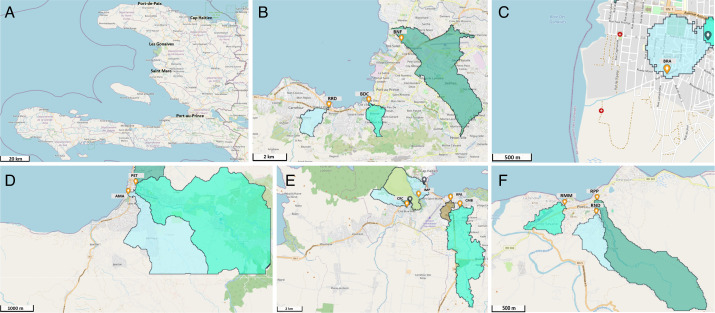
Haïti poliovirus environmental surveillance sampling sites during 2020-2023, highlighting outlined watershed boundaries for each sampling site (blue and green shading). (**A**) Location of the cities of Port-de-Paix, Cap Haïtien, Gonaïves, Saint Marc, and Port-au-Prince. (**B**) Port-au-Prince (BDC, Bois de Chêne; BNF, Bois de Neuf; and RRD, Route Rails Diquini); (**C**) Gonaïves (BRA, Boulevard de l’Avenir); (**D**) Saint Marc (AMA, Avenue Maurepas; and PET, Rue Pétion); (**E**) Cap Haïtien (CRC, Ruelle Caporis; IMP, Impasse Pétion; CMB, Rivière Commerce Bridge; and RPA, Ruelle Patience); (**F**) Port-de-Paix (RMM, Rue Monfort Montacher; RND, Rue Notre Dame; and RPP, Rivière Port-de-Paix). Maps were generated using ES.World, a web-based geospatial platform developed by Novel-T with funding from the Gates Foundation. The platform is currently managed by the World Health Organization’s GIS Centre for Health (© WHO, 2025). Used with permission.

During the surveillance period (2020–2023), ES for PVs was conducted at 13 sampling sites distributed across five cities (Table 1; Fig. 1). Among them were 10 sites in four cities previously described during earlier surveillance periods (32, 35). In 2023, the ES network was expanded to an additional city, Port-de-Paix (PDP), because of its historical association with the 2000–2001 cVDPV1 outbreak, suboptimal vaccination coverage, and according to GPEI guidelines (36, 38). The expansion included three additional sites: Rue Monfort Montacher (RMM), Rue Notre Dame (RND), and Rivière Port-de-Paix (RPP) from PDP, which were identified through a combination of field assessments and geospatial analysis. Sampling site maps were generated using the ES maps provided by Novel-t (https://es.world), incorporating catchment area delineation, elevation, and hydrology data from a 30 m terrain model (39). Geographic coordinates were recorded for each site using a handheld GPS device (Montana 600; Garmin International, Olathe, KS), and site photographs were taken. Watershed population estimates were derived from the WorldPop spatial demographic data set (Department of Geography and Environment, University of Southampton, Southampton, United Kingdom) (39). Although some sampling sites served populations below the 100,000-threshold recommended by GPEI, they were retained due to limited safe and accessible wastewater collection points in the target cities (38).

**TABLE 1:** Haïti poliovirus environmental surveillance sites evaluated during 2020–2023, site details, including geographic coordinates, estimated watershed populations, total sampling events, and site status^*a*^

City	Site code	Total sampling events	Geographic coordinates	Estimated watershed population	Additional site details	Status
Port-au-Prince (PAP)	BNF	44	18.5815, −72.3291	418,970^*b*^	(32, 35)	Retained
	BDC	45	18.5383, −72.3539	217,902^*b*^	(32, 35)	Retained
	RRD	44	18.5345, −72.3843	92,616^*b*^	(32, 35)	Retained
Gonaïves (GON)	BRA	45	19.4383, −72.6896	22,676^*b*^	(32, 35)	Retained
Cap Haïtien (CAP)	CRC	30	19.7336, −72.2178	11,553^*b*^	(32, 35)	Terminated
	RPA	46	19.7383, −72.1844	12,151^*b*^	(32, 35)	Retained
	IMP	46	19.7405, −72.2088	35,735^*b*^	(32, 35)	Retained
	CMB	46	19.7336, −72.1772	36,134^*b*^	(32, 35)	Retained
Saint Marc (SAM)	AMA	45	19.1059, −72.7022	31,340^*b*^	(32, 35)	Retained
	PET	34	19.1102, −72.6984	79,419^*b*^	(32, 35)	Terminated
Port-de-Paix (PDP)	RMM	11	19.9366, −72.8283	17,045	Open canal on Rue Monfort Montacher	Under evaluation
	RND	11	19.9395, −72.8385	20,854	Open canal on Rue Notre Dame, arrondissement de Port-de-Paix	Under evaluation
	RPP	11	19.9411, −72.8280	23,957	Open canal between the two bridges in St. Louis (rue Dessalines and rue Sténio Vincent)	Under evaluation

^*a*^ Port-au-Prince: BNF, Bois de Neuf; BDC, Bois de Chêne; RRD, Route Rails Diquini. Gonaïves: BRA, Boulevard de l’Avenir. Cap Haïtien: CRC, Ruelle Caporis; RPA, Ruelle Patience; IMP, Impasse Pétion; CMB, Rivière Commerce Bridge. Saint Marc: AMA, Avenue Maurepas; PET, Rue Pétion. Port-de-Paix: RMM, Rue Monfort Montacher; RND, Rue Notre Dame; RPP, Rivière Port-de-Paix.

^*b*^ Updated from previously published figures (32, 35).

### Sample collection and frequency

From 2020 to 2023, CDC-Atlanta and PAHO conducted annual webinars with the Laboratoire National de Santé Publique (LNSP) and MSPP for site performance reviews and refresher training on sample collection. During the reporting period, sessions were held virtually due to travel restrictions caused by the COVID-19 pandemic and civil unrest in Haïti.

**Fig 2:**
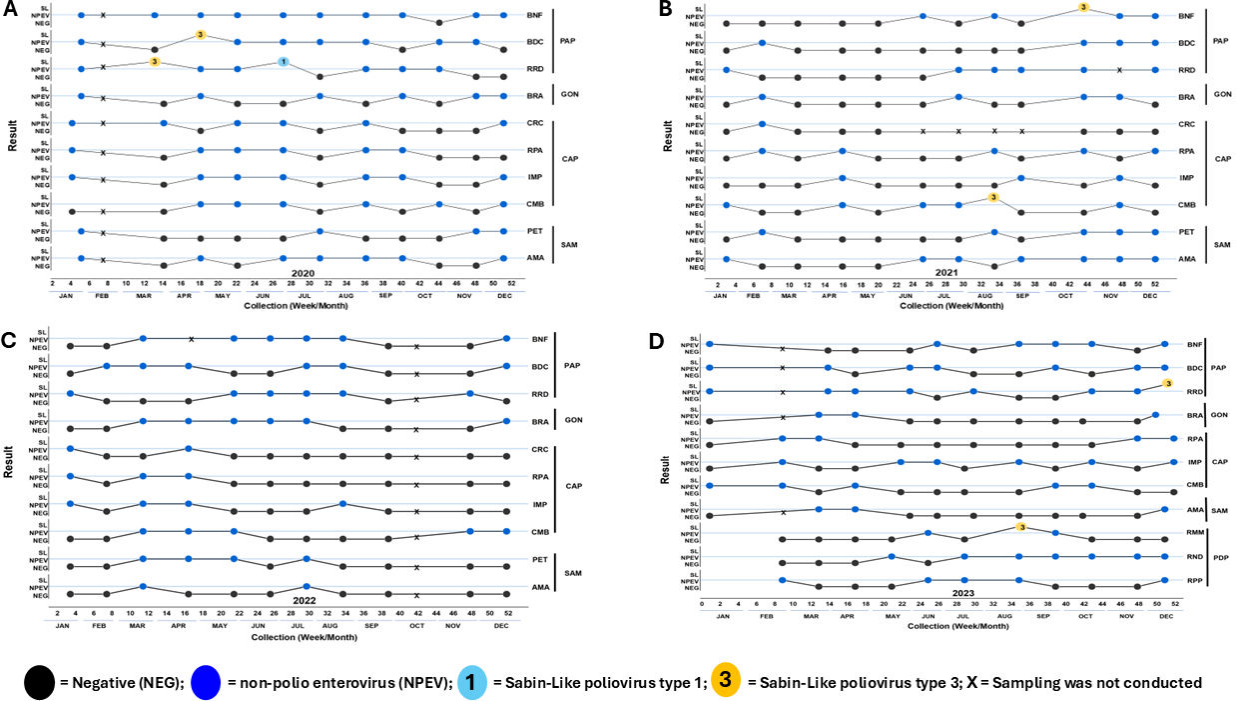
Polioviruses and enteroviruses isolated in Haïti during poliovirus environmental surveillance in all 13 sampling sites between 2020 and 2023 by year, epidemiological week, and sampling site. (**A**) ES results in 2020 (10 sites, 11 sampling events [SE] per site; no sampling at any site in February); (**B**) ES results in 2021 (10 sites, SE varies per site: 12 at most sites, 11 at RRD, and 8 at CRC); (**C**) ES results in 2022 (10 sites, SE varies per site: 11 at most sites, 10 at BNF; sampling was not conducted at any site in October and at the BNF site in April due to travel difficulties); (**D**) ES results in 2023 (11 sites, SE varies per site: 12 at RPA, IMP, and CMB sites, 11 at all other sites; sampling was not conducted at BNF, BDC, RRD, BRA, and AMA in February due to civil unrest. Port-au-Prince (PAP): BNF, Bois de Neuf; BDC, Bois de Chêne; RRD, Route Rails Diquini. Gonaïves (GON): BRA, Boulevard de l’Avenir. Cap Haïtien (CAP): CRC, Ruelle Caporis; RPA, Ruelle Patience; IMP, Impasse Pétion; CMB, Rivière Commerce Bridge. Saint Marc (SAM): PET, Rue Pétion; AMA, Avenue Maurepas. Port-de-Paix (PDP): RMM, Rue Monfort Montacher; RND, Rue Notre Dame; and RPP, Rivière Port-de-Paix.

Collection of wastewater samples (1 L) from active ES sites was conducted at intervals of approximately 4 weeks during January 2020 to December 2023 (48 months) across the five cities: Port-au-Prince (PAP) [three sites, 133 total sampling events], Gonaïves (GON) [one site, 45 events], Cap Haïtien (CAP) [four sites, 168 events], Saint Marc (SAM) [two sites, 79 events], and Port-de-Paix (PDP) [three sites, 33 events] (Table 1; Fig. 2).

Samples were collected using the grab sampling method with a swing sampler (NASCO, Fort Atkinson, WI) connected to a 1 L Nalgene bottle (Cole-Parmer, Vernon Hills, IL). Grab samples are single-point collections representing the source’s wastewater composition at the time and location of sampling; the source’s composition may not be constant over time and with water flow changes throughout the day. For each collection, the time, date, sample temperature, and weather conditions on the day of collection and the previous day were recorded. Samples were stored at 2°C–8°C immediately after collection until arrival at LNSP, where they were stored at −20°C until they were shipped frozen on dry ice to CDC-Atlanta.

### Pre-analytical processing

At CDC’s Poliovirus and Picornavirus Branch (PPB), sample characteristics (temperature, condition, and arrival time) were recorded upon receipt, and samples were stored at −20°C until analysis. Each 1 L sample was thawed at 22°C for 24 h before processing.

### Concentration of environmental samples

The 458 samples, collected from January 2020 to December 2023, were processed using the “concentration and filtration elution” (CaFÉ) method (40). Briefly, magnesium chloride (2.5 M; EMD Millipore Corp, Burlington, MA) was added to 500 mL of wastewater, and the pH was adjusted to 3.5. The sample was filtered through a series of 5 and 0.45 µm negatively charged filters (Advantec, Toyo Roshi Kaisha, Ltd., Uchisaiwaicho, Chiyoda City, Japan). Filters containing trapped viruses were subsequently cut into pieces and eluted twice with beef extract (3% wt/vol, Criterion, Hardy Diagnostics, Santa Maria, CA); the resulting eluate (15–18 mL) was treated with chloroform (20% vol/vol; Sigma-Aldrich, St. Louis, MO) to obtain concentrates of approximately 10–13 mL.

### Virus isolation and molecular characterization

Three milliliters (mL) of concentrate were inoculated into six 25 cm^2^ flasks: 0.5 mL each into five flasks with confluent monolayers of L20B cell line (recombinant murine cells that express human PV receptor) to amplify PVs specifically, and 0.5 mL inoculated into one flask of RD cell line (human rhabdomyosarcoma cells) to allow amplification of any culturable enteroviruses following the WHO isolation protocols (41). Cytopathic effect-positive virus isolates were screened by real-time reverse transcription-PCR using the intratypic differentiation (ITD) assay kit for the presence of enteroviruses (EV assay) and typing of all three PV serotypes and genotypes (42–44). Any Sabin-like type 1 (SL1) and 3 (SL3) were confirmed through additional PCR testing that distinguished them from VDPV strains (45). Briefly, 1 µL of virus isolate was tested in a 20 µL total reaction volume using 10 µL XLT Toughmix enzyme (Quanta Bioscience, Beverly, MA), 8 µL water, and 1 µL primer and probe mix for each assay. Both ITD and VDPV assay kits were supplied by the International Reagent Resource (IRR) through ATCC (Manassas, VA), a contracting agency to CDC.

### ES site performance assessment

ES sites were assessed annually by calculating the proportion of sampling events within a calendar year that yielded the isolation of any enterovirus, including both PVs and non-polio enteroviruses, presented as a percentage of the annual enterovirus isolation rate. During the annual surveillance period, a site’s performance was deemed adequate if the annual enterovirus isolation rate was ≥50%. Decisions to terminate a site also considered factors such as canal water stagnation caused by trash accumulation, dryness, and a change to the sample collector’s safety.

## RESULTS

### Sample collection analyses

From January 2020 through December 2023, wastewater was collected across five cities in Haïti: PAP, GON, CAP, SAM, and PDP. Sampling typically occurred between median times of 6:39 a.m. and 9:16 a.m. (Table S1). Rainfall was documented on the day prior to sampling in 0%–11% and on the day of sampling in 0%–9% of sampling events, respectively. The median laboratory-measured pH values ranged from 6.4 to 7.2 across all sites (Table S1).

### Enterovirus detection

#### Port-au-Prince

**Fig 3:**
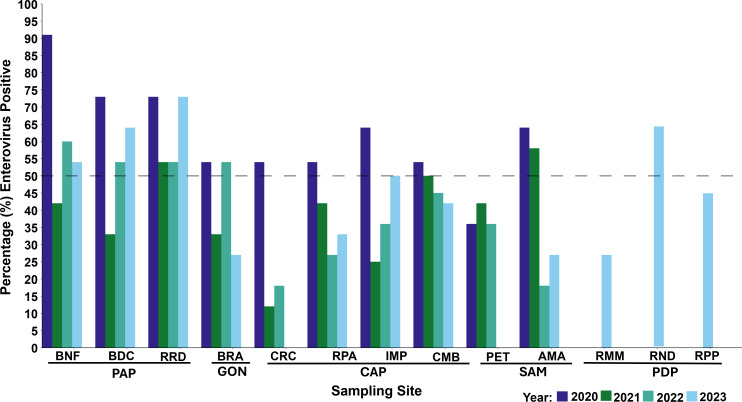
Percentage (%) of enteroviruses detected across all 13 sampling sites in Haïti from 2020 to 2023. The dotted line depicts the site performance indicator for the enterovirus detection rate at 50%. Port-au-Prince (PAP): BNF, Bois de Neuf; BDC, Bois de Chêne; RRD, Route Rails Diquini. Gonaïves (GON): BRA, Boulevard de l’Avenir. Cap Haïtien (CAP): CRC, Ruelle Caporis; IMP, Impasse Pétion; RPA, Ruelle Patience; CMB, Rivière Commerce Bridge. SAM: PET, Rue Pétion; AMA, Avenue Maurepas. Port-de-Paix (PDP): RMM, Rue Monfort Montacher; RND, Rue Notre Dame; RPP, Rivière Port-de-Paix. CRC and PET, sites terminated in January 2023; RMM and RND, sites added in February 2023.

A total of 133 wastewater samples were collected from three sites in PAP: Bois de Chêne (BDC), Bois de Neuf (BNF), and Route Rails Diquini (RRD). SL3 PV was isolated from BDC and BNF in one collection event in 2020 and 2021, respectively. At the RRD site, SL1 and SL3 PVs were isolated from two collection events in 2020, and SL3 was isolated in one collection event in 2023. Annual enterovirus isolation rates varied across the three sites, ranging from 73% to 91% in 2020, 33% to 54% in 2021, 54% to 60% in 2022, and 54% to 73% in 2023 (Table 1; Fig. 2 and 3; Fig. S1).

#### Gonaïves

Forty-five samples were collected from the Boulevard de l'Avenir (BRA) site. No SL PVs were isolated over the 4 years. Annual enterovirus detection rates at BRA were 54% in 2020 and 2022, 33% in 2021, and 27% in 2023 (Table 1; Fig. 2 and 3; Fig. S1).

#### Cap Haïtien

A total of 168 samples were collected from four sites—Ruelle Caporis (CRC), Ruelle Patience (RPA), Impasse Pétion (IMP), and Rivière Commerce Bridge (CMB). SL3 PV was detected once at CMB in 2021; no additional SL PVs were isolated during the surveillance period. Annual enterovirus isolation rates across CAP sites ranged from 54% to 64%, 12% to 50%, 18% to 45%, and 33% to 50% in 2020, 2021, 2022, and 2023, respectively. Due to sub-optimal enterovirus detection rates in 2021 and 2022 and site dryness from June to September 2021, collection at the CRC site was terminated in January 2023 (Table 1; Fig. 2 and 3; Fig. S1).

#### Saint Marc

In Saint Marc, 79 samples were collected from two sites—Rue Pétion (PET) and Avenue Maurepas (AMA). No SL PVs were detected throughout the 4 years. Annual enterovirus detection rates at PET ranged from 36% to 42%, while at AMA, they ranged from 16% to 64%. The PET site was terminated in January 2023 following a review of annual enterovirus detection rates (Table 1; Fig. 2 and 3; Fig. S1).

#### Port-de-Paix

In Port-de-Paix, 33 samples were collected from three sites—RMM, RND, and RPP between February 2023 and December 2023 (11 months). SL3 PV was detected in a sample collected from the RMM site in August 2023. Enterovirus detection rates during this period were 27% at RMM, 64% at RND, and 45% at RPP. These three sites remained under evaluation for performance after the surveillance period reported here (Table 1; Fig. 2 and 3; Fig. S1).

## DISCUSSION

This report presents findings from ES for PVs in 458 wastewater samples collected approximately monthly from five cities in Haïti between January 2020 and December 2023. No WPVs or VDPVs were detected during the surveillance period.

Haïti’s national routine immunization schedule includes one dose of inactivated PV vaccine (IPV) at 6 weeks of age and three doses of bivalent OPV (bOPV containing serotypes 1 and 3) at birth, 10, and 14 weeks, and a booster at 9 months (46–48). Following OPV administration, the Sabin and SL vaccine virus are shed into feces, enabling their detection in wastewater samples through ES (49–51). The population Pol3 vaccine coverage (the percentage of children who received the third dose of any polio-containing vaccine, either OPV or IPV) remained relatively stable, 51% from 2019 to 2023 (46).

In 2020 and 2021, SL PVs were intermittently detected in PAP (BDC, BNF, and RRD sites) and at CAP (CMB site). The PV detections in PAP over time may reflect its larger catchment population, increasing the odds of an immunization event and detection of viral shedding in the catchment area (Table 1) (35). However, in 2022, no SL PVs were detected from any sampling site, concurrent with lower enterovirus detection rates (18%–60%) compared to 2020 (36%–91%) (Fig. 2). This decline might be caused by environmental factors, such as poor sample quality, collection challenges, and water flow obstructions (i.e., water stagnation due to trash accumulation in the sampling canals) limiting overall enterovirus detection (including PV) (19, 22, 52, 53). In 2023, SL3 PV was detected in two sampling events at the RMM site in PDP and at the RRD site in PAP. Although these detections confirm viral shedding, the RMM site’s smaller catchment population (~17,000) compared to RRD (~92,600) may reduce the odds of detection because of a lower probability that a child in the catchment area was recently vaccinated, was shedding virus in stool, and defecated during a sample collection window. In contrast, larger catchments provide a greater chance of capturing at least one excreter, despite the increased sample dilution (Table 1; Fig. 2) (54).

Overall, SL PV detection through virus isolation was lower during the 2020–2023 surveillance period, with only seven SL PV isolates, compared to 24 isolates detected over 46 months during 2016–2019, a period that coincided with OPV vaccination campaigns (32, 35). This decline likely reflects the low routine immunization coverage in the country during 2020–2023, which limited the number of people shedding virus into the environment (55). In addition, the absence of supplemental OPV (bOPV1 and 3) immunization activities (i.e., national immunization days) during the 2020–2023 surveillance period might have contributed to this decline.

From 2020 to 2023, 49 AFP cases were notified in Haïti, with only one AFP case in 2021 with SL viruses isolated from their stool (SL PV types 1 and 3) (G. Rey-Benito, personal communication). The non-polio AFP case detection rate is a key indicator of AFP surveillance sensitivity. A well-performing AFP surveillance system should detect at least one non-polio AFP case for every 100,000 children under 15 years annually. However, the annual rate in Haïti from 2020 to 2023 remained below the recommended standard of one, with values between 0.27 and 0.40, indicating suboptimal AFP surveillance (56, 57). Given the suboptimal performance, ES in Haïti provides a critical supplementary approach for detecting PV transmission in the absence of AFP cases.

**Fig 4:**
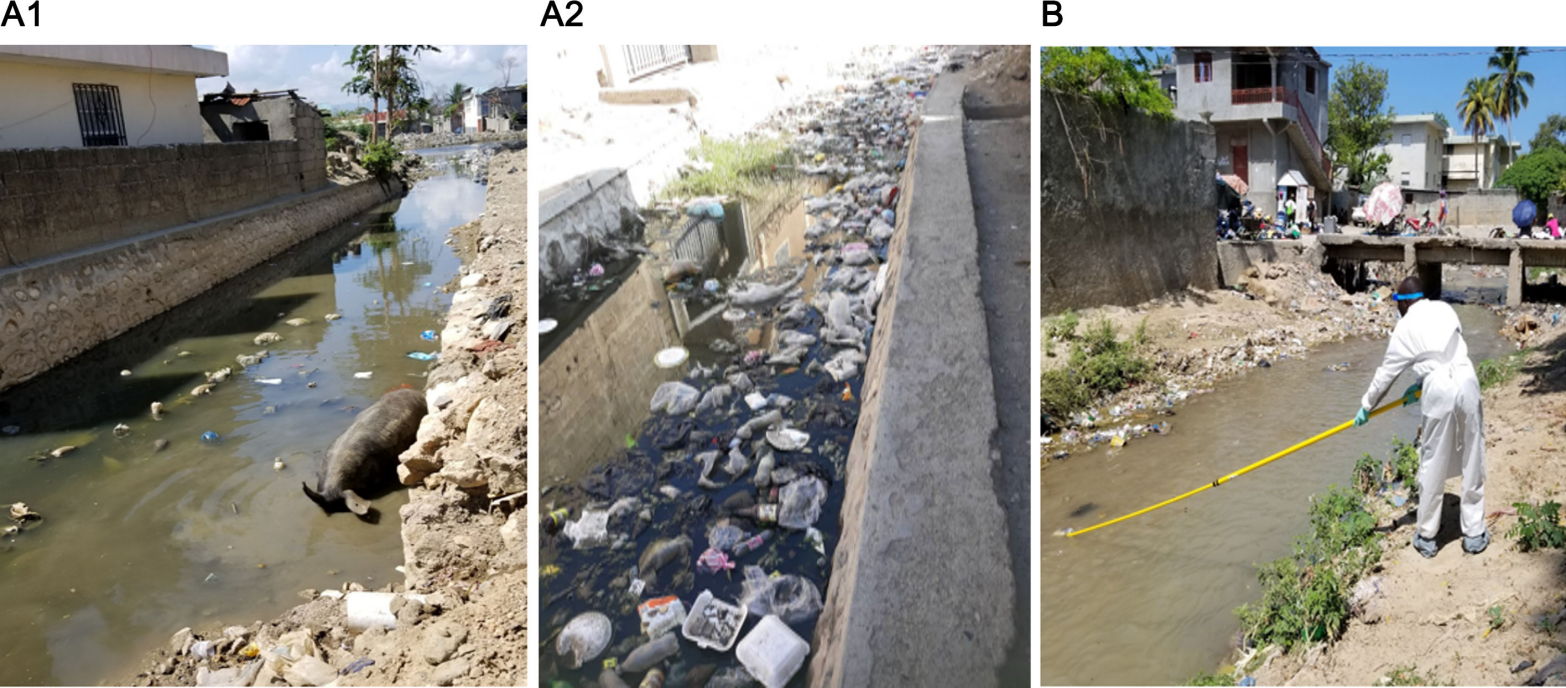
Photographs of two environemental surveillance sampling sites in Haïti, terminated in January 2023. (**A1**) Ruelle Caporis (CRC) site in Cap Haïtien (CAP) during a site assessment in 2019, showing flowing water in the canal; (**A2**) same site (CRC) in 2022, a debris-filled and stagnant canal following reduced water flow; (**B**) Rue Pétion (PET) site in Saint Marc (SAM), showing a collector using a swing-sampler for wastewater sample collection in 2022.

The isolation of enteroviruses from wastewater serves as a performance indicator for PV ES sites. A site is considered to perform adequately if ≥50% of samples collected over a 6-to-12-month period yield enterovirus detection during monthly sampling (21, 37, 38). In January 2023, the collection at two sites was terminated; the CRC site in CAP due to prolonged canal dryness rendering wastewater collection impossible, and at the PET site in Saint Marc due to persistently suboptimal annual enterovirus detection rates (36% in 2020 and 2022; 42% in 2021). These lower rates might have been caused by water stagnation in the canal with significant trash accumulation over the collection period that impaired access to the sampling site (Fig. 2 to 4).

To expand ES for PVs in Haïti geographically to the *Nord-Ouest* (Northwest) department, additional sampling sites were established in PDP (RMM, RND, and RPP) in February 2023, following a joint site assessment by the LNSP and Direction d’Épidémiologie, de Laboratoire et de Recherche. This decision was based on the area’s involvement in the cVDPV1 outbreak, where the first case of AFP caused by a cVDPV1 in Haïti was detected in 2000 (34). The Global Polio Surveillance Action Plan 2022–2024 prioritized improving the quality of ES sites worldwide through standardized site assessments, sampling protocols, and laboratory support, aligning with Haïti’s efforts to enhance site performance and resource optimization (58).

Annual enterovirus detection rates during this surveillance period ranged from 16% to 91%, slightly lower than in the 2016–2019 period (25%–100%), and are consistent with findings from other low- and middle-income countries, including Nigeria and Sierra Leone (32, 35, 59, 60). Most sampling events occurred before 9:00 a.m., aligning with peak sewage flow times to optimize virus recovery (14, 54). Laboratory sample conditions remained stable, with pH values consistently in the neutral range (Table S1).

There are several limitations to conducting ES in Haïti during the surveillance period. The political instability, insecurity, the COVID-19 pandemic in 2020, the cholera outbreak in 2022, and natural disasters, such as the 2021 earthquake and a hurricane, hindered routine annual site evaluations by CDC-Atlanta and PAHO staff and delayed sample shipment to CDC-Atlanta (61–65). Despite these numerous challenges, nearly all monthly wastewater collection was maintained throughout the surveillance period, highlighting the commitment of national public health authorities in Haïti.

The implementation of ES for PVs in Haïti reflects a proactive approach to strengthening eradication efforts in vulnerable settings. The absence of WPVs and VDPVs is encouraging, and ongoing investment in ES infrastructure at high-performing sites, improved site selection, and regular site visits are crucial elements for maintaining high-quality ES. Any ES activities should be paired with improved immunization coverage and enhanced AFP surveillance, critical to sustaining Haïti’s PV-free status and supporting global eradication goals.

## SUPPLEMENTAL MATERIAL

Fig. S1 aem.01179-25-s0001.tif Enteroviruses isolated in Haiti during poliovirus environmental surveillance 2020–2023.

Table S1 aem.01179-25-s0002.tif Selected water quality measurements, time of day of collection, and general weather conditions for each site.

ASM does not own the copyrights to Supplemental Material that may be linked to, or accessed through, an article. The authors have granted ASM a non-exclusive, world-wide license to publish the Supplemental Material files. Please contact the corresponding author directly for reuse.
