# Supplementary figures and images for: Tracking poliovirus through wastewater: environmental surveillance insights from Haïti (2020–2023)
Source: Appl Environ Microbiol. 2025 Nov 18;91(12):e01179-25. doi: 10.1128/aem.01179-25 (PMC12724337; doi:10.1128/aem.01179-25)

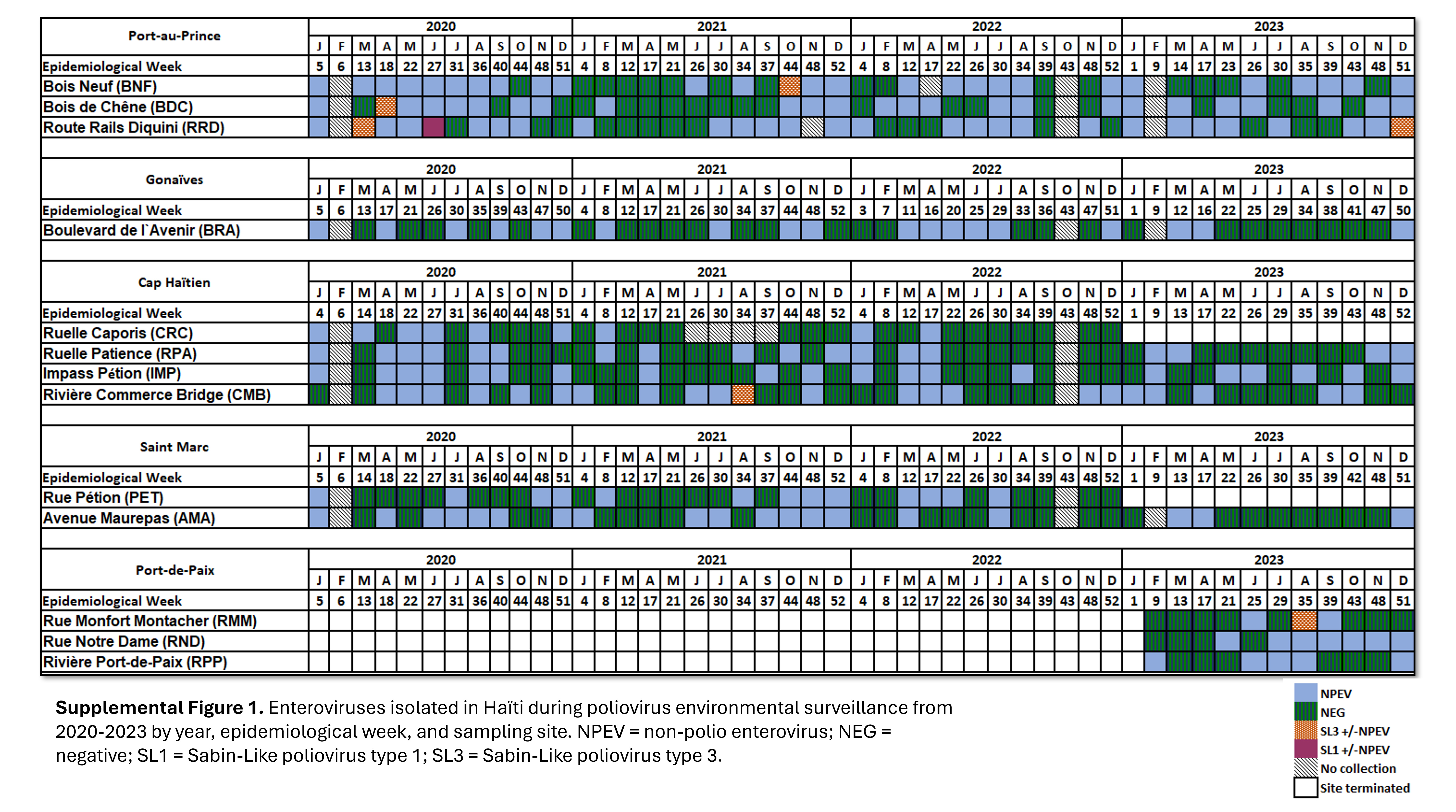

Supplement: Fig. S1 — Enteroviruses isolated in Haiti during poliovirus environmental surveillance 2020–2023. [file aem.01179-25-s0001.tif]

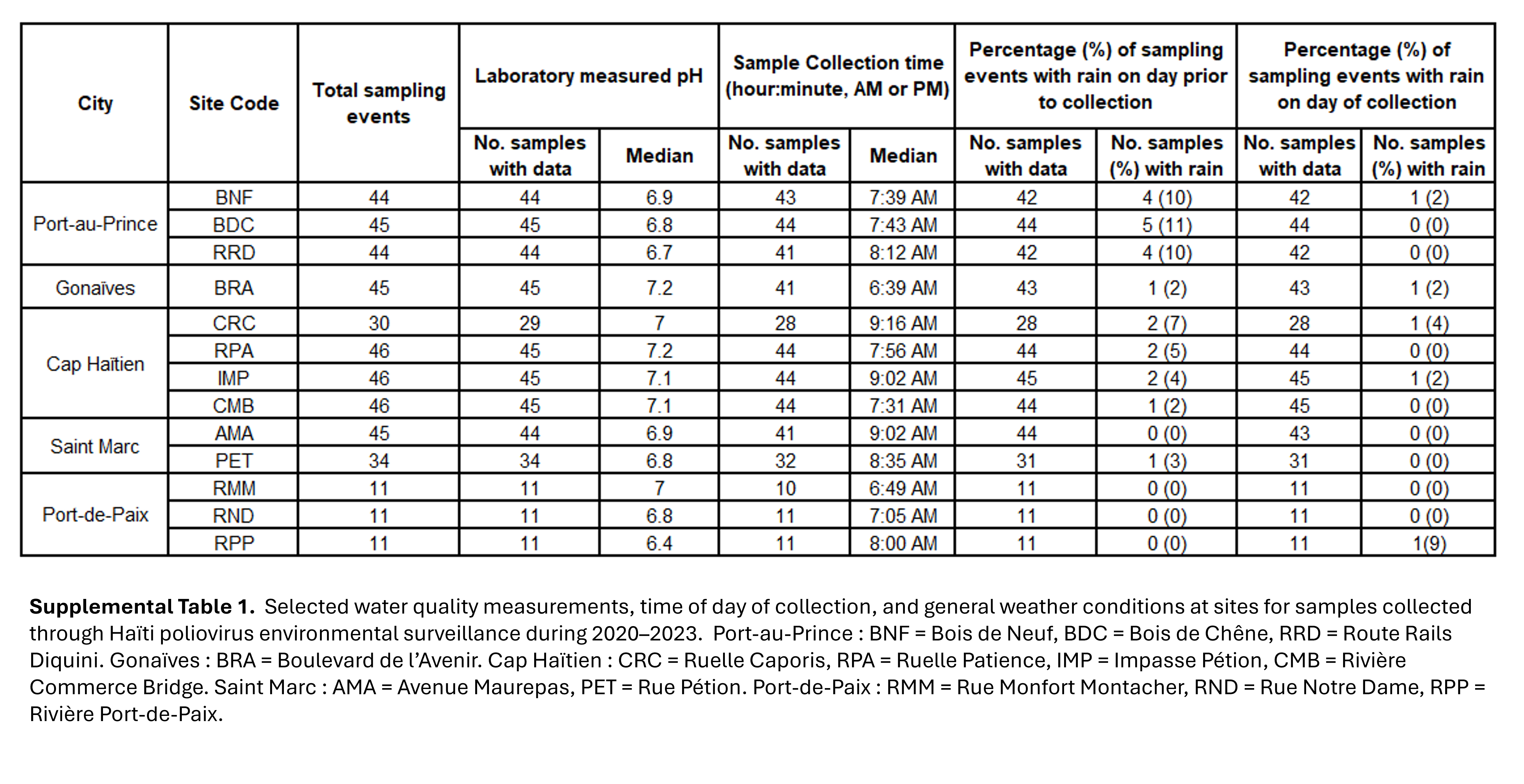

Supplement: Table S1 — Selected water quality measurements, time of day of collection, and general weather conditions for each site. [file aem.01179-25-s0002.tif]
